# Risk factors for electrocardiographic left ventricular hypertrophy in a young Chinese general population: the Hanzhong adolescent cohort study

**DOI:** 10.1186/s12872-021-01966-y

**Published:** 2021-03-31

**Authors:** Yue-Yuan Liao, Ke Gao, Bo-Wen Fu, Lei Yang, Wen-Jing Zhu, Qiong Ma, Chao Chu, Yu Yan, Yang Wang, Wen-Ling Zheng, Jia-Wen Hu, Ke-Ke Wang, Yue Sun, Chen Chen, Jian-Jun Mu

**Affiliations:** 1 Department of Cardiovascular Medicine, First Affiliated Hospital of Xi’an Jiaotong University, Xi’an, People’s Republic of China; 2 Key Laboratory of Molecular Cardiology of Shaanxi Province, Xi’an, Shaanxi People’s Republic of China; 3 The Second Affiliated Hospital of Xi’an Jiaotong University, Xi’an, People’s Republic of China; 4 Department of Cardiology, First Affiliated Hospital of Medical School, Xi’an Jiaotong University, 277 Yanta West Street, Xi’an, 710061 People’s Republic of China

**Keywords:** Electrocardiogram, Cornell voltage-duration product, Risk factors, Left ventricular hypertrophy, Cohort study

## Abstract

**Background:**

Electrocardiographic left ventricular hypertrophy (ECG-LVH) is a common manifestation of preclinical cardiovascular disease. The present study aimed to investigate risk factors for ECG-LVH and its prevalence in a cohort of young Chinese individuals.

**Methods:**

(1) A total of 1515 participants aged 36–45 years old from our previously established cohort who were followed up in 2017 were included. Cross-sectional analysis was used to examine risk factors for ECG-LVH and its prevalence. (2) A total of 235 participants were recruited from the same cohort in 2013 and were followed up in 2017. Longitudinal analysis was used to determine the predictors of LVH occurrence over the 4-year period. We used multivariable logistic regression models to calculate OR and 95% CIs and to analyze risk factors for ECG-LVH.

**Results:**

In the cross-sectional analysis, the prevalence of LVH diagnosed by the Cornell voltage-duration product in the overall population and the hypertensive population was 4.6% and 8.8%, respectively. The logistic regression results shown that female sex [2.611 (1.591–4.583)], hypertension [2.638 (1.449–4.803)], systolic blood pressure (SBP) [1.021 (1.007–1.035)], serum uric acid (SUA) [1.004 (1.001–1.006)] and carotid intima-media thickness (CIMT) [67.670 (13.352–342.976)] were significantly associated with the risk of LVH (all *P* < 0.05). In the longitudinal analysis, fasting glucose [1.377 (1.087–1.754)], SBP [1.046 (1.013–1.080)] and female sex [1.242 (1.069–1.853)] were independent predictors for the occurrence of LVH in the fourth year of follow-up.

**Conclusions:**

Our study suggested that female sex, hypertension, SBP, SUA and CIMT were significantly associated with the risk of LVH in young people. In addition, fasting glucose, SBP and female sex are independent predictors of the occurrence of LVH in a young Chinese general population.

**Supplementary Information:**

The online version contains supplementary material available at 10.1186/s12872-021-01966-y.

## Background

Left ventricular hypertrophy (LVH), as defined by electrocardiography (ECG) or echocardiographic criteria, is a potential independent predictor of cardiovascular morbidity and mortality [1, 2]. The mechanism of LVH development is not fully understood, but hemodynamic factors, such as increased afterload and activation of the renin–angiotensin–aldosterone system in the context of hypertension [3, 4], are important for the development of LVH. Blood pressure is the strongest independent risk factor for LVH, and LVH has been observed in all stages of hypertension [5]. However, LVH can also be present in normotensive subjects, and the severity of hypertension is far from explaining the changes in left ventricular mass index (LVMI) [6]. Thus, nonhemodynamic mechanisms, such as metabolic factors and genetic factors, are likely to contribute to the development of LVH.

Nonhaemodynamic factors are likely to be involved in the pathogenesis of LVH, as increased blood pressure values explain less than 30% of variations in LVMI, both in normotensive and hypertensive subjects [7]. Some risk factors, such as age, sex [8], body mass index (BMI) [9], SUA [10], insulin resistance and diabetes mellitus [11, 12], may all play roles in the pathogenesis of LVH. However, in China and other countries, most studies on LVH have mainly select middle-aged and elderly patients with hypertension, with a large age range and a large number of complications [2, 4, 5, 8–12]. At present, there are no reports on the prevalence and related risk factors of LVH among young subjects in large population-based samples, especially in the rural young population of China. According to China Hypertension Survey 2012–2015 [13], among Chinese adults ≥ 18 years old, the overall crude prevalence of hypertension was 27.9%, and the weighted prevalence was 23.2%. The prevalence of hypertension among young people aged 18–24, 25–34 and 35–44 years old was 4.0%, 6.1% and 15.0%, respectively, which increased compared with the Chinese hypertension survey in 2002 [14], and the prevalence of LVH will continue to increase in the future. Therefore, it is of great significance to identify the prevalence and related risk factors of LVH in young subjects.

Therefore, we conducted cross-sectional and longitudinal analyses based on our previously established cohort to investigate related risk factors for ECG-LVH and its prevalence in a cohort of young Chinese individuals from the general population.

## Methods

### Subjects

The study population was derived from the Hanzhong Adolescent Hypertension Study, which was established in 1987. The Hanzhong Adolescent Hypertension Study is an ongoing prospective, population-based cohort study of 4623 Chinese adolescents who regularly undergo follow-ups to investigate the development of cardiovascular risk factors originating in children and young adults. Details of the study protocol have been published elsewhere [15–17].

**Fig. 1:**
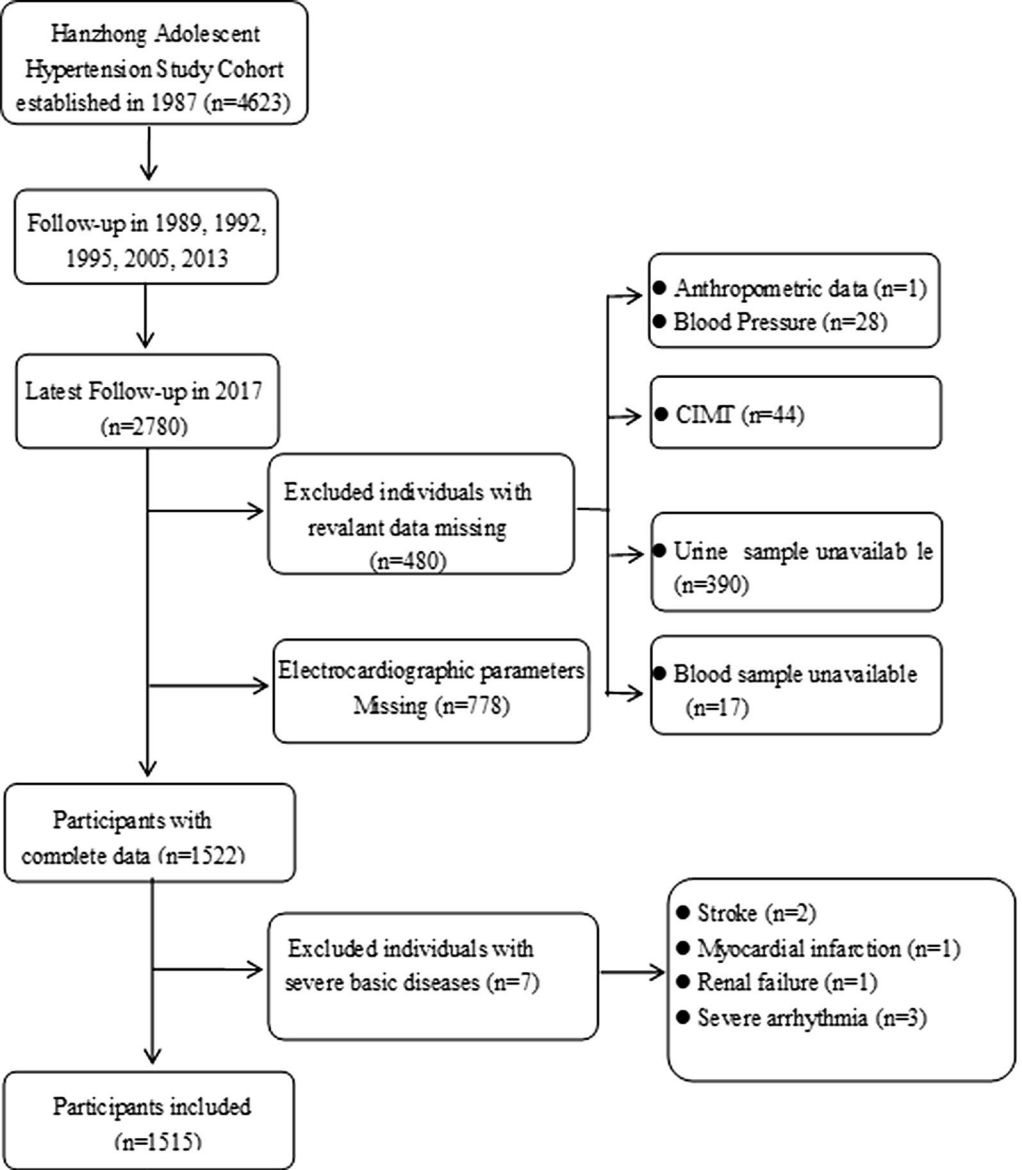
Flow diagram for participants in cross-sectional study

This study was divided into two sections: (1) In the cross-sectional analysis, we used data from the large cohort that was followed up in 2017. The participant selection process is described in Fig. 1. Participants were excluded if they had missing data on ECG parameters (n = 778), relevant measurement data (n = 480) and a self-identified history of stroke, coronary heart disease, renal failure or severe arrhythmia (n = 7), leaving 1515 subjects for the primary analyses. (2) In the longitudinal analysis, we used data from a small cohort of 338 subjects that was created based on the large cohort in 2005. The detailed study design and procedures have been published previously [17, 18]. We followed up with this small cohort in 2013 and 2017. For the current analysis, we did not measure ECG parameters or blood biochemical indicators in 2005, so the small cohort that was followed up in 2013 was considered the baseline for investigating risk factors for ECG-LVH. In 2013, we performed echocardiographic examination in this small cohort. Methods and criteria of echocardiographic LVH were described in detail elsewhere [12]. Participants who were lost to follow-up in 2013 and 2017 (n = 100) and those with echocardiographic LVH in 2013 (n = 3) were excluded. The remaining 235 subjects with complete data in 2013 and 2017 were included in the longitudinal analysis.

This study was approved by the Ethics Committee of the First Affiliated Hospital of Xi’an Jiaotong University, and written informed consent was obtained from each participant at each examination phase. The study complied with the principles of the Helsinki Declaration. Trial registration Number: NCT02734472. Date of registration: 12/04/2016.

### Anthropometric measurements

Information on occupation, education, smoking habits, alcohol consumption, physical activity, medical conditions and medication use was collected using a self-administered questionnaire. Body weight and height were measured, and BMI was calculated as weight (kg) divided by height (m^2^). Blood pressure was measured with the participant in a seated position using a standard mercury sphygmomanometer as previously described [15, 19]. Hypertension was defined as a systolic blood pressure (SBP) ≥ 140 mmHg or a diastolic blood pressure (DBP) ≥ 90 mmHg or the use of antihypertensive agents according to the subjects’ self-report or pharmacy data. Carotid intima-media thickness, defined as the distance from the intima-luminal interface to the media-adventitial interface, was measured as previously described [17]. Three images were obtained for both common carotid arteries, and the average of the six that were measured were used for analysis. The same sonographer who was blinded to the subjects’ clinical status carried out all the measurements.

### Biochemical assays

Venous blood samples were obtained from all participants after a 12-h overnight fast. Standardized measurements for fasting glucose, serum total cholesterol, triglycerides, low-density lipoprotein cholesterol (LDL-C), high-density lipoprotein cholesterol (HDL-C), serum creatinine, SUA, high-sensitivity C-reactive protein (hs-CRP), urinary creatinine and albumin were performed in the clinical laboratory at the First Affiliated Hospital of Xi’an Jiaotong University. Details of these assays have been described previously [15, 16, 18]. The estimated glomerular filtration rate (eGFR) was calculated using the formula adapted from the Modification of Diet in Renal Disease equation on the basis of data from Chinese subjects with CKD [16, 20]. The urinary albumin-to-creatinine ratio (uACR) was calculated as urine albumin in mg divided by urine creatinine in mmol (mg/mmol).

### Electrocardiographic measurements

According to the Minnesota code criteria, standard supine 12-lead electrocardiograms (ECGs) were recorded, and LVH was defined by the ECG Cornell voltage-duration product [RaVL + SV3 (adjusted by the addition of 8 mm for women) × QRS duration] > 2440 mm ms [21, 22], using the mean value of three consecutive measurements. Two observers performed measurements in duplicate with a graduated lens on standard 12-lead ECGs registered at a 25 mm/s speed and 10 mm/mV gain. The R wave amplitude in aVL and V_5_ and the S wave depth in V_1_ and V_3_ were measured as the distance (mm) between the isoelectric line and the nadir or zenith, respectively. The QRS duration was the average of three consecutive measurements of the QRS complexes. The QT interval and the QT interval duration corrected for the previous cardiac cycle length (QTc) were calculated as described in detail elsewhere [22]. Other ECG criteria, such as Sokolow-Lyon voltage, Sokolow-Lyon voltage-duration product and Cornell voltage criteria, were calculated according to the ECG parameters.

### Statistical analysis

Data are expressed as medians (inter-quartile range) for non-normally distributed values, as the means ± standard deviations for normally distributed values, and as percentages. Significant differences between the groups were calculated using the Student’s t-test, the Mann–Whitney test and *χ*^2^-test as appropriate. The correlation coefficient *r* was measured to assess the relationship between two variables. We analyzed the association between related factors and LVH with logistic regression in cross-sectional analysis and longitudinal analyses. All statistical analyses were conducted using SPSS 25.0 (SPSS, Inc., Chicago, IL). *P* < 0.05 was considered statistically significant.

## Results

### Characteristics of participants in the cross-sectional study

Table 1 presents the characteristics of all subjects according to LVH status. Among this young population, the average of Cornell voltage duration product criteria was 1201.45 ± 494.86 mm ms, and a total of 70 subjects had LVH according to Cornell voltage duration product criteria. The prevalence of LVH diagnosed by the Cornell voltage-duration product in the total population and the hypertensive population was 4.6% and 8.8%, respectively. The proportion of females and hypertension, BMI, waist-hip ratio (WHR), SBP, DBP, SUA, uACR and CIMT were higher in participants with LVH than in those without LVH. There were no statistically significant differences in age, heart rate, eGFR, blood lipids, fasting glucose and urinary uric acid/creatinine ratio (uUA/Cre) between the two groups.

**Table 1:** Characteristics of participants categorized by LVH status (n = 1515)

Variable	ALL	Subjects with LVH	Non-LVH	*P* values
No. of subjects	1515	70	1445	*–*
Age, y	43 (40, 45)	43 (41, 45)	43 (40, 45)	0.493
Female, no. (%)	663(43.8)	36 (51.4)	627 (43.4)	0.021
BMI, Kg/m^2^	23.7 (21.8*–*25.8)	25.2 (22.7*–*26.8)	23.6 (21.8*–*25.8)	0.004
WHR	0.92 (0.86*–*0.96)	0.96 (0.89*–*0.99)	0.92 (0.87*–*0.97)	0.012
Current smoking, no. (%)	724 (47.8)	30 (42.9)	694 (48.0)	0.453
alcohol use, no. (%)	474 (31.3)	19 (27.1)	455 (31.5)	0.438
Hypertension, no. (%)	171 (11.3)	15 (21.4)	156 (10.8)	0.002
Diabetes mellitus, no. (%)	103 (6.8)	4 (5.7)	99 (6.9)	0.662
SBP, mmHg	121.0 (112.0*–*130.7)	128.7 (114.7*–*138.8)	120.0 (112.0*–*129.3)	0.001
DBP, mmHg	75.3 (68.7*–*82.7)	82.9 (72.9*–*89.4)	75.3 (68.7*–*82.7)	< 0.001
Heart rate, beats/min	73.0 (67.0*–*79.0)	74.0 (67.8*–*83.0)	73.0 (66.0*–*79.0)	0.133
SUA, μmol/L	268.1 (210.7*–*323.1)	280.3 (224.1*–*352.0)	260.9 (205.1*–*320.6)	0.043
Serum creatinine, umol/L	76.02 ± 14.56	74.78 ± 15.16	76.04 ± 14.55	0.473
eGFR, mL/min/1.73m^2^	92.11 ± 26.07	88.60 ± 17.58	92.18 ± 26.20	0.255
Fasting glucose, mmol/L	4.57 (4.28*–*4.89)	4.53 (4.20*–*4.93)	4.57 (4.27*–*4.89)	0.725
Total cholesterol, mmol/L	4.51 (4.04*–*5.00)	4.60 (4.02*–*4.99)	4.50 (4.04*–*5.00)	0.962
Triglycerides, mmol/L	1.36 (0.95*–*1.91)	1.41 (0.97*–*1.86)	1.35 (0.95*–*1.91)	0.860
LDL-C, mmol/L	2.51 (2.14*–*2.89)	2.50 (2.04*–*2.95)	2.50 (2.14*–*2.89)	0.710
HDL-C, mmol/L	1.15 (0.99*–*1.33)	1.15 (1.00*–*1.31)	1.15 (0.99*–*1.33)	0.911
uUA/Cre	0.19 (0.10*–*0.32)	0.22 (0.13*–*0.41)	0.19 (0.11*–*0.32)	0.069
UACR, mg/g	8.30 (5.45*–*14.42)	10.90 (7.09*–*24.83)	8.26 (5.39*–*14.21)	0.001
hs-CRP, mg/L	0.31 (0.15*–*0.74)	0.32 (0.15*–*0.77)	0.30 (0.15*–*0.75)	0.847
CIMT, mm	0.62 (0.53*–*0.75)	0.74 (0.60*–*0.83)	0.62 (0.53*–*0.75)	< 0.001
Cornell index, mm ms	1201.45 ± 494.86	3010.62 ± 665.11	1167.40 ± 422.50	< 0.001

Normally distributed variables are expressed as mean ± SD as determined by the Student’s t-test, non-normally distributed variables are expressed as medians (inter-quartile range) as determined by Mann–Whitney test, categorical variables are expressed as numbers and percentages by *χ*^2^-test

*BMI* body mass index, *WHR* waist hip rate, *SBP* systolic blood pressure, *DBP* diastolic blood pressure, *SUA* serum uric acid, *eGFR* estimated glomerular filtration rate, *LDL-C* low-density lipoprotein, *HDL-C* high-density lipoprotein, *uUA/Cre* urinary uric acid/creatinine ratio, *uACR* urinary albumin-to-creatinine ratio, *hs-CRP* high-sensitivity C-reactive protein, *CIMT* carotid intima-media thickness

Additional file 1: Table S1 presents the ECG parameters of all subjects according to LVH status. Sokolow-Lyon voltage, Sokolow-Lyon voltage duration product, Cornell voltage criteria, Cornell voltage duration product, QRS duration and QTc were higher in participants with LVH than in those without LVH.

### Associations of various characteristics with Cornell index

**Fig. 2:**
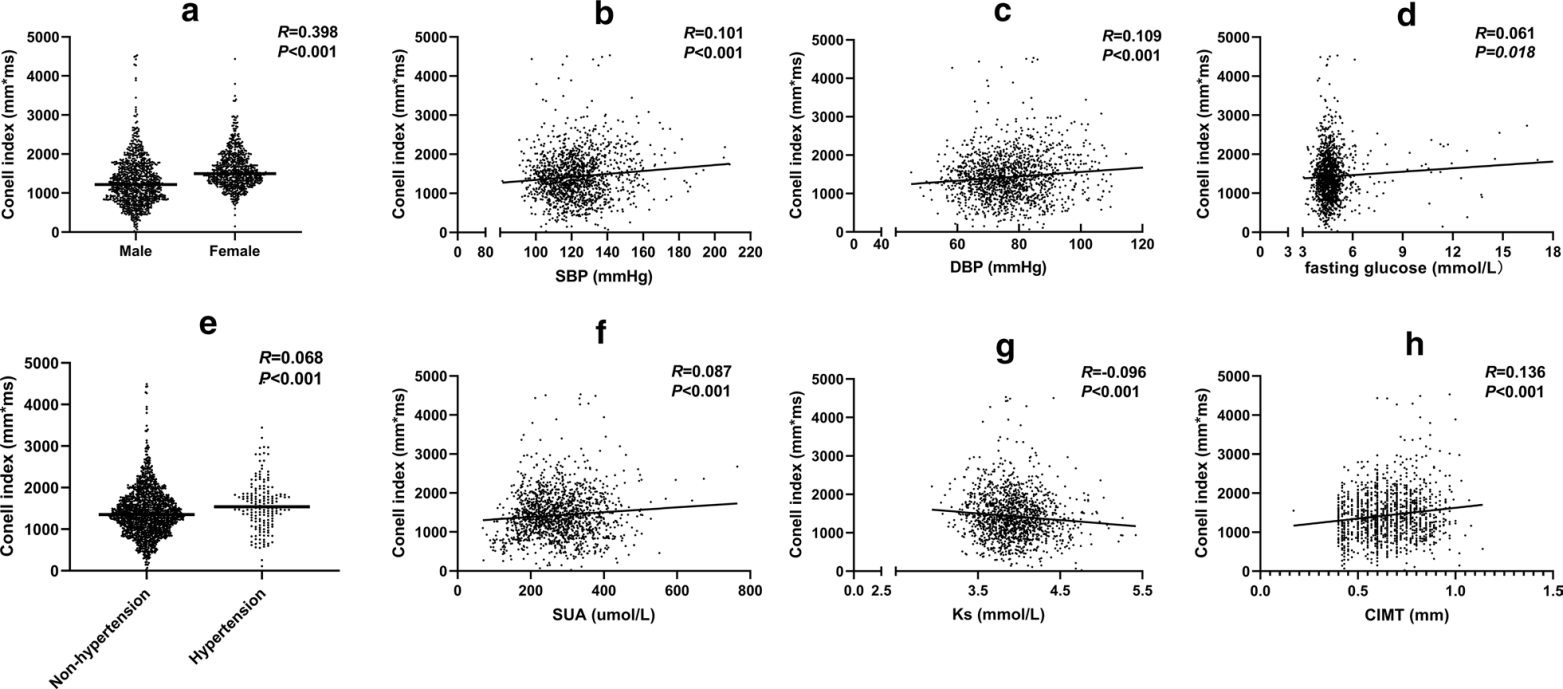
Relationship between various characteristics and Cornell index by correlation analysis. *R*, correlation coefficient. *DBP* diastolic blood pressure, *Ks* serum potassium, *CIMT* Carotid intima-media thickness, *SBP* systolic blood pressure, *SUA* serum uric acid

The correlation analyses showed that Cornell index was positively correlated with female sex (r = 0.398, *P* < 0.001) and hypertension (r = 0.068, *P* < 0.001), SBP (r = 0.101, *P* < 0.001) and DBP (r = 0.109, *P* < 0.001). In addition, LVH was also associated with fasting glucose (r = 0.061, *P* = 0.018), SUA (r = 0.087, *P* < 0.001) and CIMT (r = 0.136, *P* < 0.001), while it was inversely associated with serum potassium (r = -0.096, *P* < 0.001) (Fig. 2).

To further comprehensively and accurately investigate this finding, we analyzed the relationship between various characteristics and LVH based on logistic regression in a cross-sectional study. We found that female sex [2.611 (1.591–4.583)], hypertension [2.638 (1.449–4.803)], SBP [1.021 (1.007–1.035)], SUA [1.004 (1.001–1.006)] and CIMT [67.670 (13.352–342.976)] were significantly associated with LVH (all *P* < 0.05) (Table 2). DBP (*P* = 0.114), BMI (*P* = 0.537), WHR (*P* = 0.430), fasting glucose (*P* = 0.198) and serum potassium (*P* = 0.215) did not remain significant in the model. To exclude the effect of collinearity between variables on the results, we performed a collinearity diagnosis of variables using variance inflation factor (VIF) values. As the results are shown, we can consider that there is no collinearity between the variables (Additional file 1: Table S2).

**Table 2:** Association between various characteristics and the risk of LVH by logistic regression analysis (n = 1515)

Variable	Odds ratios (confidence interval)	*P* values
Sex (female)	2.611 (1.591*–*4.583)	< 0.001
Age, y	1.002 (0.917*–*1.094)	0.969
Hypertension	2.638 (1.449*–*4.803)	0.005
Diabetes mellitus	0.880 (0.249*–*3.116)	0.843
BMI, kg/m^2^	1.029 (0.939*–*1.128)	0.537
WHR	5.431 (0.081*–*364.747)	0.430
SBP, mmHg	1.021 (1.007*–*1.035)	0.003
DBP, mmHg	1.014 (0.997*–*1.032)	0.114
Fasting glucose, mmol/L	1.114 (0.945*–*1.313)	0.198
Serum potassium, mmol/L	0.614 (0.285*–*1.326)	0.215
eGFR, mL/min/1.73m^2^	0.999 (0.989*–*1.010)	0.878
SUA, umol/L	1.004 (1.001*–*1.006)	0.013
Total cholesterol, mmol/L	0.685 (0.230*–*2.040)	0.497
Triglycerides, mmol/L	1.061 (0.678*–*1.659)	0.796
LDL-C, mmol/L	1.029 (0.309*–*3.426)	0.963
HDL-C, mmol/L	3.869 (0.664*–*22.557)	0.133
CIMT, mm	67.670 (13.352*–*342.976)	< 0.001

Logistic regression analyses were used to test the risk of LVH. Age, sex, hypertension, diabetes, smoking status, alcohol consumption, BMI, WHR, SBP, DBP, fasting glucose, SUA, eGFR, total cholesterol, triglycerides, LDL-C, HDL-C, uUA/Cre and CIMT were all included in the model

To extend this finding to clinical practice, we further investigated the association between various characteristics and LVH based on logistic regressions in normotensive subjects (n = 1344). Female sex [1.366 (1.210–1.636)], SBP [1.017 (1.003–1.031)], SUA [1.003 (1.001–1.006)] and CIMT [47.068 (7.946–278.798)] were still significantly associated with LVH (Additional file 1: Table S3).

### Participant characteristics in the longitudinal study

The characteristics of those who participated in both surveys are presented in Table 3. Subjects had higher WHR, heart rate, serum creatinine, fasting glucose, total cholesterol, LDL-C, uACR and CIMT; lower DBP, SUA and HDL-C levels; and a higher proportion of alcohol consumption and diabetes after a 4-year follow-up. There were no statistically significant differences in BMI, hypertension rate, SBP, eGFR or triglycerides between baseline and follow-up. In the fourth year of follow-up, there were 26 subjects with new-onset LVH.

**Table 3:** Characteristics of the study participants at baseline and follow-up (n = 235)

Variable	Baseline in 2013	Follow-up in 2017	*P* values
Female, no. (%)	104 (44.2)	104 (44.2)	–
Age, years	39 (36, 41)	43 (40, 45)	< 0.001
Current smoking, no. (%)	110 (41.5)	105 (44.6)	0.489
Alcohol use, no. (%)	23 (9.1)	65 (27.8)	< 0.001
BMI, Kg/m^2^	23.83 (21.63*–*26.33)	23.73 (22.19*–*25.92)	0.826
WHR	0.90 (0.85*–*0.95)	0.93 (0.87*–*0.97)	< 0.001
Hypertension, no. (%)	79 (29.7)	53 (22.6)	0.792
Diabetes mellitus, no. (%)	6 (2.3)	15 (6.2)	0.023
SBP, mmHg	120.7 (111.3*–*132.0)	121.0 (112.8*–*131.7)	0.403
DBP, mmHg	80.0 (72.7*–*89.0)	76.3 (69.3*–*84.8)	0.001
Heart rate, beats/min	70.0 (66.0*–*80.0)	74.0 (67.0*–*81.0)	0.022
SUA, μmol/L	300.9 (246.0*–*369.7)	267.9 (209.7*–*335.8)	< 0.001
Serum creatinine, umol/L	74.21 ± 14.02	80.82 ± 17.69	< 0.001
eGFR, mL/min/1.73m^2^	94.90 ± 21.13	94.12 ± 26.23	0.694
Fasting glucose, mmol/L	4.58 (4.26*–*4.92)	5.00 (4.69*–*5.41)	< 0.001
Total cholesterol, mmol/L	4.27 (3.83*–*4.72)	4.51 (4.07*–*4.99)	< 0.001
Triglycerides, mmol/L	1.42 (1.00*–*2.07)	1.37 (0.98*–*1.96)	0.405
LDL-C, mmol/L	2.33 (2.00*–*2.70)	2.51 (2.14*–*2.89)	0.001
HDL-C, mmol/L	1.66 (1.43*–*1.87)	1.14 (1.01*–*1.32)	< 0.001
UACR, mg/g	6.07 (4.08*–*10.72)	8.80 (5.77*–*15.17)	< 0.001
CIMT, mm	0.50 (0.40*–*0.60)	0.64 (0.55*–*0.74)	< 0.001
LVH, no. (%)	*–*	26 (11.0)	*–*

*BMI* body mass index, *WHR* waist hip rate, *SBP* systolic blood pressure, *DBP* diastolic blood pressure, *SUA* serum uric acid, *eGFR* estimated glomerular filtration rate, *LDL-C* low-density lipoprotein, *HDL-C* high-density lipoprotein, *uACR* Urinary albumin-to-creatinine ratio, *CIMT* carotid intima-media thickness

### Predictors of LVH in the fourth year of follow-up

Traditional risk factors at baseline in regard to LVH in the fourth year of follow-up in logistic regression analysis are shown in Table 4. Three factors were identified in the binary logistic regression analysis as predictors of fourth-year LVH. These factors were fasting glucose [1.377 (1.087–1.754)], SBP [1.046 (1.013–1.080)] and female sex [1.242 (1.069–1.853)].

**Table 4:** Independent predictors of left ventricular hypertrophy (LVH) in the fourth year in logistic regression analysis (n = 235)

Baseline characteristic	Odds ratios (confidence interval)	*P* values
Sex (female)	1.242 (1.069*–*1.853)	0.043
Age, y	0.872 (0.675*–*1.126)	0.293
Hypertension	0.242 (0.041*–*1.418)	0.116
Diabetes mellitus	0.810 (0.07*–*1.874)	0.054
BMI, kg/m^2^	1.042 (0.821*–*1.323)	0.736
WHR	6.321 (0.012*–*34.795)	0.194
SBP, mmHg	1.046 (1.013*–*1.080)	0.006
DBP, mmHg	0.979 (0.883*–*1.084)	0.678
Fasting glucose, mmol/L	1.377 (1.087*–*1.754)	0.008
eGFR, mL/min/1.73m^2^	0.981 (0.948*–*1.017)	0.298
SUA, umol/L	0.991 (0.980*–*1.002)	0.100
Total cholesterol, mmol/L	2.118 (0.058*–*7.240)	0.683
Triglycerides, mmol/L	0.828 (0.4.4*–*1.695)	0.605
LDL-C, mmol/L	0.267 (0.004*–*1.704)	0.534
HDL-C, mmol/L	0.313 (0.006*–*15.257)	0.558
CIMT, mm	97.040 (0.081*–*1156.760)	0.205

Logistic regression analyses were used to test the independent predictors of LVH, age, sex, hypertension, diabetes, BMI, WHR, SBP, DBP, fasting glucose, SUA, eGFR, total cholesterol, triglycerides, LDL-C, HDL-C and CIMT were all included in the model

## Discussion

Electrocardiographic left ventricular hypertrophy is a common manifestation of preclinical cardiovascular disease. Nonhaemodynamic factors are likely to be involved in the pathogenesis of LVH, and the severity of hypertension is far from explaining the changes in left ventricular mass. With this prospective cohort study, we were the first to investigate risk factors for ECG-LVH and its prevalence in a cohort of young Chinese individuals. We found that the prevalence of LVH diagnosed by the Cornell voltage-duration product in the overall population and the hypertensive population was 4.6% and 8.8%, respectively. Our study suggested that female sex, hypertension, SBP, SUA and CIMT were significantly associated with the risk of LVH in young people. In addition, fasting glucose, SBP and female sex are independent predictors of the occurrence of LVH in a young Chinese general population.

LVH appears to be highly prevalent in individuals with hypertension but also in the general population. Currently, there are no large epidemiological studies on the LVH detection rate and its risk factors in young Chinese populations. Several previous studies have investigated the prevalence of LVH in the general population. For example, Joji Ishikawa et al. [23] found that in 10,755 individuals from the general Japanese population, the detection rate of LVH diagnosed by the Cornell voltage-duration product was 6.4% in all subjects and 11.0% in the hypertension subgroup. Lehtonen AO et al. [24] found that the LVH detection rates according to the Cornell voltage criteria in individuals with normal blood pressure, hypertension grade 1 and hypertension grade 2 among the 5800 Finnish population were 5.1%, 9.0% and 13.1%, respectively. In line with these two studies, we found that the prevalence of LVH diagnosed by the Cornell voltage-duration product in the total young population and hypertensive population were 4.6% and 8.8%, respectively.

Currently, the mechanism of LVH development is not completely clear, except for hemodynamics, and its pathogenesis is related to age, sex, body mass, race, genetic factors, metabolic status (such as insulin resistance and hyperuricemia) and other factors [8–12]. To the best of our knowledge, this is the first study to investigate risk factors for ECG-LVH in a cohort of young Chinese individuals from the general population. We observed that fasting glucose, SBP and female sex are independent predictors of the occurrence of LVH in the fourth year of follow-up. Female, higher fasting glucose and SBP can increase the risk of LVH. Blood pressure is the strongest independent risk factor for LVH, and that approximately 30% of hypertensive patients may have LVH, and the detection rate of LVH is positively correlated with the severity of hypertension. [24] Blood glucose is closely associated with insulin levels, and increased glucose levels may cause hyperinsulinemia. Our observation is similar to that of Lin et al., who observed that baseline fasting glucose is correlated with the 4-year change in LVMI and is an independent predictor for LVMI and the occurrence of LVH after a 4-year follow-up in normotensive healthy elderly subjects without diabetes mellitus [25].Insulin itself could induce cardiovascular hypertrophy by acting on insulin growth factor receptors and simulating cell proliferation and lipid deposition [26, 27]. In addition, our results showed that SUA and CIMT were significantly associated with the risk of LVH in young people.

Yoshio Iwashima et al. [28] showed that SUA is independently associated with LVMI and suggest that hyperuricemia combined with LVH is an independent and powerful predictor for Cardiovascular disease in asymptomatic subjects with essential hypertension. In line with this study, we found that SUA was significantly associated with LVH in correlation and logistic regression analyses in this Chinese cohort. In addition, many studies have shown that SUA may impair NO generation, induce endothelial dysfunction, and promote oxidative metabolism and smooth muscle cell proliferation [28, 29], which are known to induce cardiac hypertrophy. These results suggest that cardiac hypertrophy may be partially attributable to an increase in UA itself. Similar to what we found, Nam-ho Kim et al. [30] conducted a cross-sectional study with 9266 middle-aged and elderly individuals from the general populations in Korea and found that compared with other quartile groups, the risk of LVH increased by 48% in the highest quartile of CIMT. Carotid IMT thickening was associated with the presence of endothelial dysfunction, oxidative stress, inflammatory mediators and metabolic factors, which can lead to myocardial collagen hyperplasia and fibrosis aggravation and may thereby promote LVH [30, 31].

In our study, we also found that sex was significantly associated with LVH in the young population. Numerous studies have confirmed that there are sex differences in LVH, and the prevalence of LVH in females was higher than that in males, regardless of the use of ECG or echocardiography. A retrospective analysis of 30 studies showed that the prevalence of LVH diagnosed by ECG in hypertensive patients was 35.6%-40.9%, among which the detection rates were 36.0%-43.5% in males and 37.9%-46.2% in females [32]. A study also showed that the prevalence of LVH in Chinese hypertension patients diagnosed by echocardiography was significantly higher in females than in males [33]. Currently, the mechanism of the correlation between sex and LVH has not been fully elucidated but may be related to ECG diagnostic methods [21, 22], sex differences in the size and mass of the left ventricle and its response to chronic pressure load [34, 35], and sex hormones factors [35].

Some limitations of our study merit consideration. First, our results were obtained from young northern Chinese individuals and consequently may not be generalizable to other age and ethnic groups with different demographics. Second, we used electrocardiograms to diagnose LVH at the follow-up in 2017 and failed to conduct echocardiography. Echocardiographic detection of LVH provides more accurate estimates of LVMI than ECGs. Although echocardiography is the preferred method to diagnose LVH in clinical practice, ECGs are commonly used as the first-line instrument for detecting LVH due to its convenience, cost-effectiveness, good reproducibility and availability in large cohort settings. Finally, some participants were lost during the follow-up period, and the number of subjects with LVH during the follow-up period was relatively small. However, as we know, the subjects of this study are young individuals from the general population who are currently in the period of subclinical target organ damage, and the prevalence of hypertension and cardiovascular disease is relatively low. Clarifying the risk factors for Cardiovascular disease in young populations is of great significance for the primary prevention of cardiovascular disease, and we will continue to follow up.

In conclusion, our study shows female sex, hypertension, SBP, SUA and CIMT were significantly associated with the risk of LVH in the young Chinese population. AND fasting glucose, SBP and female sex are independent predictors of the occurrence of LVH in the fourth year of follow-up. Our study suggests that, even in an apparently healthy, relatively young population, in addition to the active control of blood pressure, regular monitoring of SUA and fasting glucose and the lowering of UA and glucose if necessary, may be actively considered to reduce the risk of left ventricular hypertrophy.

## Supplementary Information

**Additional file 1.**: **Table S1**. ECG parameters of participants categorized by LVH status (n=1515). **Table S2**. The collinearity diagnosis analysis between variables (n=1515). **Table S3**. Association between various characteristics and the risk of LVH by multiple logistic regression analysis in normotensive subjects (n=1344).

## Data Availability

The datasets generated and/or analyzed during the current study are not publicly available due the ongoing nature of this study, but are available from the corresponding author on reasonable request.

## Abbreviations

BMI: Body mass index
CIMT: Carotid intima-media thickness
ECG-LVH: Electrocardiographic left ventricular hypertrophy
eGFR: Estimated glomerular filtration rate
HDL-C: High-density lipoprotein cholesterol
hs-CRP: High-sensitivity C-reactive protein
LVH: Left ventricular hypertrophy
LDL-C: Low-density lipoprotein cholesterol
LVMI: Left ventricular mass index
SBP: Systolic blood pressure
SUA: Serum uric acid
uACR: Urinary albumin-to-creatinine ratio
WHR: Waist-hip ratio
