# Supplementary material for: Risk factors for electrocardiographic left ventricular hypertrophy in a young Chinese general population: the Hanzhong adolescent cohort study
Source: BMC Cardiovasc Disord. 2021 Mar 31;21:159. doi: 10.1186/s12872-021-01966-y (PMC8011382; doi:10.1186/s12872-021-01966-y)
Supplement: Supplementary file 1 — Additional file 1.: Table S1. ECG parameters of participants categorized by LVH status (n=1515). Table S2. The collinearity diagnosis analysis between variables (n=1515). Table S3. Association between various characteristics and the risk of LVH by multiple logistic regression analysis in normotensive subjects (n=1344). [file 12872_2021_1966_MOESM1_ESM.doc]

**Risk factors for electrocardiographic** **left ventricular hypertrophy** **in a young Chinese general population: the Hanzhong Adolescent Cohort Study**

**Short Title: Risk factors for ECG-LVH in young population**

Yue-Yuan Liao^1,2†^, Ke Gao^1,2†^, Bo-Wen Fu^1,2^, Lei Yang^1,2^, Wen-Jing Zhu^3^, Qiong Ma^1,2^, Chao Chu^1,2^, Yu Yan^1,2^, Yang Wang^1,2^, Wen-Ling Zheng^1^, Jia-Wen Hu^1^, Ke-Ke Wang^1,2^, Yue Sun^1,2^, Chen Chen^1,2^, Jian-Jun Mu^1,2^*.

^1^ Department of Cardiovascular Medicine, First Affiliated Hospital of Xi'an Jiaotong University, Xi’an, China

^2^ Key Laboratory of Molecular Cardiology of Shaanxi Province, Xi’an, Shaanxi, P.R. China

^3^ The Second Affiliated Hospital of Xi 'an Jiaotong University

† These authors contributed equally to this work.

^*^Corresponding author: Department of Cardiology, First Affiliated Hospital of Medical School, Xi’an Jiaotong University, 277 Yanta West Street, Xi’an, 710061, China

E-mail: [mujjun@163.com](mailto:mujjun@163.com)

Phone: +86-29-85323524;

Fax: +86-29-85323804.

**Table S1**. ECG parameters of participants categorized by LVH status (n=1515)

| Characteristics | ALL | Subjects with LVH | Non-LVH | *P* values |
| --- | --- | --- | --- | --- |
| Sokolow- Lyon voltage, mV | 1.95±0.57 | 2.21±1.01 | 1.94±0.56 | <0.001 |
| Sokolow- Lyon voltage-  duration product, mm·ms | 1970.05±67.90 | 2518.46±145.58 | 1959.73±65.19 | <0.001 |
| Cornell voltage, mV | 0.92±0.20 | 2.34±0.78 | 0.90±0.39 | <0.001 |
| Cornell voltage-duration product , mm·ms | 1201.45±494.86 | 3010.62±665.11 | 1167.40±422.50 | <0.001 |
| QRS duration, ms | 100.34±10.12 | 111.96±16.81 | 100.12±9.83 | <0.001 |
| QT interval, ms | 386.37±26.25 | 389.16±23.52 | 386.32±26.30 | 0.371 |
| QTc interval, ms | 404.54±21.14 | 413.83±26.32 | 404.36±20.99 | 0.004 |

QTc: the QT interval duration corrected for the previous cardiac cycle length.

Table S2. The collinearity diagnosis analysis between variables (n=1515).

| Variable | Tolerance | VIF Value |
| --- | --- | --- |
| Sex (Female) | .853 | 1.173 |
| Age, y | .960 | 1.042 |
| Hypertension | .971 | 1.030 |
| Diabetes mellitus | .913 | 1.096 |
| BMI, kg/m^2^ | .608 | 1.645 |
| WHR | .636 | 1.572 |
| SBP, mmHg | .397 | 2.519 |
| DBP, mmHg | .404 | 2.477 |
| Fasting glucose, mmol/L | .899 | 1.113 |
| Serum potassium, mmol/L | .970 | 1.031 |
| eGFR, mL/min/1.73m^2^ | .925 | 1.081 |
| SUA, umol/L | .891 | 1.122 |
| Total cholesterol, mmol/L | .192 | 5.203 |
| Triglycerides, mmol/L | .327 | 3.054 |
| LDL-C, mmol/L | .202 | 4.954 |
| HDL-C, mmol/L | .395 | 2.533 |
| CIMT, mm | .954 | 1.048 |

BMI, body mass index; WHR, waist hip rate; SBP, systolic blood pressure; DBP, diastolic blood pressure; SUA, serum uric acid; eGFR, estimated glomerular filtration rate; LDL-C, low-density lipoprotein; HDL-C, high-density lipoprotein; CIMT, carotid intima-media thickness; VIF, variance inflation factor.

**Table S3**. Association between various characteristics and the risk of LVH by multiple

logistic regression analysis in normotensive subjects (n=1344)

| Variable | Odds ratios (confidence interval) | *P* values |
| --- | --- | --- |
| Sex (Female) | 1.366 (1.210-1.636) | <0.001 |
| Age, years | 1.024 (0.923-1.136) | 0.658 |
| Diabetes mellitus | 0.951 (0.212-4.260) | 0.948 |
| BMI, kg/m^2^ | 0.997 (0.889-1.118) | 0.962 |
| WHR | 108.408 (0.550-21366.310) | 0.082 |
| SBP, mmHg | 1.017 (1.003-1.031) | 0.020 |
| DBP, mmHg | 1.009 (0.989-1.030) | 0.338 |
| Fasting glucose, mmol/L | 1.131 (0.955-1.339) | 0.154 |
| Serum potassium, mmol/L | 0.515 (0.212-1.247) | 0.141 |
| eGFR, mL/min/1.73m^2^ | 1.000 (0.988-1.011) | 0.941 |
| SUA, umol/L | 1.003 (1.001-1.006) | 0.045 |
| Total cholesterol, mmol/L | 0.554 (0.172-1.785) | 0.323 |
| Triglycerides, mmol/L | 1.147 (0.733-1.793) | 0.548 |
| LDL-C, mmol/L | 1.320 (0.345-5.048) | 0.685 |
| HDL-C, mmol/L | 6.409 (0.115-36.830) | 0.057 |
| CIMT, mm | 47.068 (7.946-278.798) | <0.001 |

Logistic regression analyses were used to test the risk of LVH in normotensive subjects. Age, sex, diabetes, smoking status, alcohol consumption, BMI, WHR, SBP, DBP, fasting glucose, SUA, eGFR, total cholesterol, triglycerides, LDL-C, HDL-C and CIMT were all included in the model.
